# Prevalence trends and disease burden of diabetes and prediabetes in Chinese adults of Shanghai

**DOI:** 10.1111/1753-0407.13391

**Published:** 2023-05-18

**Authors:** Jianfeng Pei, Yanyun Li, Yihui Yang, Minna Cheng, Yan Shi, Wang Hong Xu

**Affiliations:** ^1^ Department of Epidemiology, School of Public Health Fudan University Shanghai China; ^2^ Department of Non‐communicable Disease Prevention and Control Shanghai Municipal Center for Disease Control and Prevention Shanghai China; ^3^ National Clinical Research Center for Aging and Medicine, Huashan Hospital Fudan University Shanghai China

**Keywords:** awareness, diabetes, prediabetes, prevalence, trend, 认知度, 糖尿病, 糖尿病前期, 患病率, 趋势

## Abstract

**Background:**

To estimate secular trends and disease burden of diabetes and prediabetes among Chinese adults.

**Methods:**

Three population‐based surveys were performed among Chinese adults in Shanghai in 2002–2003 (*n* = 12 302), 2009 (*n* = 7414), and 2017 (*n* = 18 960). Diabetes and prediabetes were defined using the 1999 World Health Organization (WHO) criteria. Cochran‐Armitage trend test was used to examine the trends in prevalence, awareness, and glycemic control status. Disability adjusted life years (DALYs) were estimated to evaluate the disease burden of diabetes‐related complications using the population attribution fraction approach based on published data.

**Results:**

The age‐adjusted prevalence of diabetes increased during the 15‐year period (*p* for trend <.001) and reached 23.0% (95% CI: 22.1 ~ 24.0%) in men and 15.7% (95% CI: 15.1 ~ 16.4%) among women in 2017. The prevalence of impaired glucose tolerance peaked in 2009, whereas that of impaired fasting glucose increased continuously (*p* for trend <.001). The awareness of diabetes was found to increase and the glycemic control rates decreased over the three surveys. The estimated DALYs of diabetes complications were found to have increased rapidly due to the increasing prevalence of diabetes and the decreasing glycemic control rates.

**Conclusions:**

Prediabetes and diabetes affect a considerable proportion of Chinese adults in Shanghai. Our results highlight the necessary to strengthen the community healthcare system in China to guarantee extensive management of diabetes and prediabetes.

## Graphical abstract

**Highlights**

- An upward trend was observed for age‐standardized prevalence of diabetes and prediabetes in Chinese adults over the 15‐year period.
- Despite the elevated awareness of diabetes, the disease burden of diabetes complications increased due to the increasing prevalence and decreasing glycemic control rate in the population.
- Our findings highlight the necessary to strengthen the community healthcare system in China to guarantee extensive management of diabetes and prediabetes.

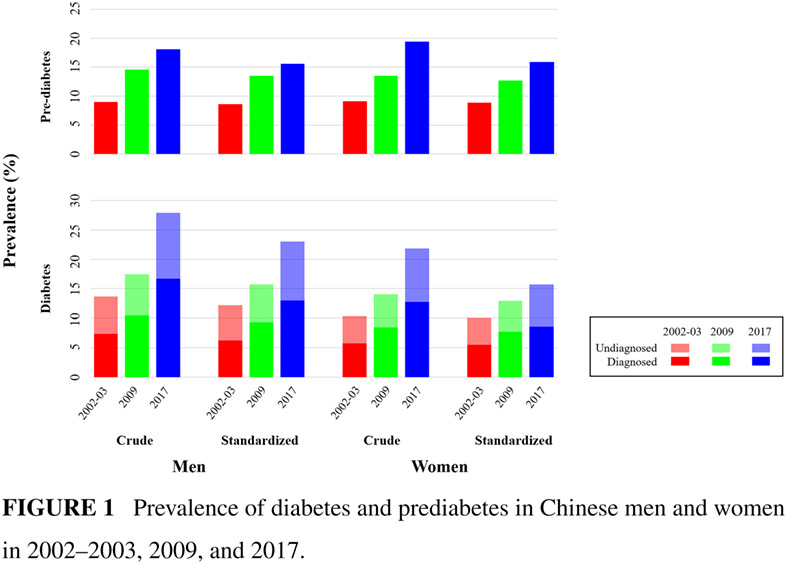

## 摘要

背景:了解中国成年人糖尿病及糖尿病前期的长期变化趋势和疾病负担。

方法:于2002—2003年, 2009年和2017年在上海市开展了3次以人群为基础的调查，分别为12 302人, 7 414人和18 960人。糖尿病和糖尿病前期的定义采用1999年WHO标准。采用Cochran‐Armitage趋势检验分析患病率, 知晓率和血糖控制状况的变化趋势。根据已发表的数据，采用人群归因分数法估算伤残调整寿命年(DALYs)，评估糖尿病相关并发症的疾病负担。

结果:15年间，年龄调整的糖尿病患病率明显增加(p for trend<0.001)，2017年男性和女性的糖尿病患病率分别达到22.0% (95%CI: 21.1~22.9%)和14.5% (95%CI: 13.9~15.2%)。糖耐量受损患病率在2009年达到峰值，空腹血糖受损患病率持续上升(p for trend <0.001)。3次调查糖尿病知晓率均有所提高，但血糖达标率均有所下降。随着糖尿病患病率的上升和血糖控制率的下降，糖尿病并发症所造成的DALYs估计值迅速增加。

结论:糖尿病前期和糖尿病在上海成年人中占有相当比例。我们的研究结果提示有必要加强社区卫生服务体系，以保证糖尿病和糖尿病前期的广泛管理。

## 1 INTRODUCTION

Diabetes (mainly type 2 diabetes) and prediabetes are metabolic disorders characterized with hyperglycemia due to insulin resistance or insufficient insulin secretion.^1^ The metabolic disorders were associated with the risk of mortality, diabetes‐related complications, and comorbidities in multiple populations,^2^, ^3^, ^4^, ^5^ resulting in immense economic burdens globally.

China has been an epidemic center of diabetes for decades.^1^ The overall prevalence in adults increased during past decades, which were 0.67% in 1981,^6^ 2.7% in 2002,^7^ 9.7% in 2008,^8^ 11.6% in 2010,^9^ 10.9% in 2013, and 12.4% in 2018.^10^ The prevalence of prediabetes, defined based on levels of fasting plasma glucose (FPG) and hemoglobin A1c (A1c), was also as high as 15.5% in 2008,^8^ 50.1% in 2010,^9^ 35.7% in 2013,^11^ and 38.1% in 2018.^10^, ^11^ These data, although from the surveys using different study designs, sampling methods, and criteria for diagnoses, illustrate a surge in prevalence and economic burdens of the diseases in the country.

In respond to the epidemic of diabetes and other noncommunicable diseases, China initiated a national health reform in 2009. Thereafter the screening and management of diabetes and prediabetes was transferred from tertiary hospitals to community healthcare centers (CHCs), greatly improving the accessibility of primary health services for patients.^12^ The effectiveness of the public health action may be demonstrated by improved awareness, diagnosis, and control rates of diabetes, particularly in Shanghai, one of the megacities in China at an advanced stage of population aging and nutritional transition.

This study was based on three population‐based surveys conducted in Shanghai, two of which have been reported in previous studies.^13^, ^14^, ^15^ This study added the latest data of the 2017 survey to evaluate secular prevalence trends and control status of diabetes and prediabetes and thus estimate diabetes‐related disease burden. The findings of the study may provide valuable insight and evidence for diabetes management in China.

## 2 MATERIALS AND METHODS

### 2.1 Study design and populations

Three population‐based surveys on diabetes were performed among residents of Shanghai, China, in 2002–2003, 2009, and 2017. The multistage sampling adopted in the surveys was previously described.^13^, ^14^, ^15^ In brief, in the 2002–2003 survey, four districts and two counties were randomly selected from a total of 12 districts and 7 counties. Then, one or two subdistricts or towns were randomly chosen from each included district or county. After that, one or two communities or villages were randomly selected from each selected subdistrict or town. Finally, 1000–2000 permanent residents between 15 and 74 years old living in the area for at least 5 years were invited for the survey. After excluding pregnant women, type 1 diabetes patients, and physically or mentally disabled individuals, a total of 17 526 eligible subjects were recruited, and 14 401 (82.2%) participated in the survey (Figure S1A).

The surveys in 2009 and 2017 followed a similar protocol. Due to the rapid urbanization of the suburbs in Shanghai; however, the sampling framework in 2017 was different from those in 2002–2003 and 2009. In addition, in the 2009 survey, only those at ages 35–74 years old were eligible, and the response rate was 62.6% (Figure S1B). In the 2017 survey, 21 625 of 23 993 eligible subjects (90.1%) completed the interview, in which 1783 subjects also participated in the 2009 survey (Figure S1C).

To make the three surveys comparable, we excluded all subjects younger than 35 years or older than 74 years. After further excluding subjects with missing values of FPG, 2‐h postprandial blood glucose (2hPG), or diagnosis of diabetes, we included 12 302 subjects (5023 men and 7279 women) of the 2002–2003 survey, 7414 subjects (3456 men and 3958 women) of the 2009 survey and 18 960 subjects (7588 men and 11 372 women) of the 2017 surveys in this analysis.

The three surveys were performed by the Shanghai Municipal Center for Disease Control and Prevention and approved by the Institutional Review Board of the center. Informed consent was obtained from each participant before data collection.

### 2.2 Data collection

The three surveys adopted a similar protocol in data collection. Trained interviewers administered a structured questionnaire at CHCs located in the residential areas of the participants. Information collected included demographic and socioeconomic factors, tobacco and alcohol use, physical activity, family history of diabetes, and diagnoses of diseases.

Body measurements were conducted for each subject in light clothing and without shoes. According to the standardized protocol, standing height and body weight were measured with stadiometer and electronic scale. Waist circumference (WC) was recorded using a cloth tape on bare skin at the midline between the lower border of the ribs and the iliac crest in the horizontal plane after a normal expiration.^16^ To examine blood pressure (BP), all subjects were advised to refrain from coffee, tea, or alcohol intake; cigarette smoking; and vigorous exercise for at least 30 min. BP was measured on the right arm in the sitting position using standard mercury sphygmomanometer after at least 5‐min rest.^17^ The interval of two BP measurements was at least 2 min and the mean was used in data analysis.^15^, ^18^, ^19^ Body mass index (BMI) was calculated as body weight in kilograms divided by standing height in meters squared (kg/m^2^).

### 2.3 Laboratory measurements

All subjects were instructed to maintain their usual physical activity and diet for 3 days before the measurements. After fasting for at least 10 h overnight, venous blood specimen was collected in a vacuum tube containing sodium fluoride for FPG measurement using glucose oxidase‐peroxidase method. For each subject without history of diabetes, an oral glucose tolerance test (OGTT) was performed using the standard 75 g‐glucose load to measure 2hPG.^15^, ^18^, ^19^, ^20^ HbA1c levels were measured via high‐performance liquid chromatography according to the recommendation of the National Glycohemoglobin Standardization Program.^21^

Non‐anticoagulated venous blood specimen was collected for the measurement of triglyceride (TG), low‐density lipoprotein cholesterol (LDLC), and high‐density lipoprotein cholesterol (HDLC). LDLC and TG levels were assessed enzymatically using commercial reagents, and HDLC was measured using phosphotungstic acid‐Mg^2+^ (PTA‐Mg) method. Any abnormal results in the assays were informed by the family doctors of subjects for early treatment.

### 2.4 Relevant definitions

Diagnosed diabetes was identified by a positive response to the question of “Have you ever been diagnosed with type 2 diabetes by a doctor?” Undiagnosed diabetes were identified according to measured glucose levels based on the 1999 criteria of the WHO.^22^ Specifically, a person can be diagnosed as type 2 diabetes when he or she has (a) a random plasma glucose ≥11.1 mmol/L accompanied by typical symptoms of diabetes such as thirst and polyuria; or (b) an FPG ≥7.0 mmol/L; or (3) a 2hPG after an OGTT ≥11.1 mmol/L.

Prediabetes referred to any participants who did not have diabetes but had an impaired fasting glucose (IFG) (FPG: 6.1–7.0 mmol/L) or impaired glucose tolerance (IGT) (2hPG after OGTT: 7.8–11.1 mmol/L).^23^ All subjects with prediabetes were further defined as with isolated‐IFG, isolated‐IGT, or both IFG and IGT.

Awareness rate was defined as the proportion of individuals with physician‐diagnosed diabetes among all patients with diabetes. Glycemic control rate was calculated as the proportion of patients with an HbA1c concentration less than 7.0% (53 mmol/mol) or FPG level less than 7.0 mmol/L among all physician‐diagnosed diabetes.^9^ Central obesity was defined as WC > 90 cm in men and > 80 cm in women according to the WHO criteria.

### 2.5 Disease burden related to glycemic status

Disability adjusted life years (DALYs) is a measure for disease burden, which was defined as summary of the number of years of healthy life lost due to premature death and disability caused by a particular disease or health condition. In this study, DALYs by diabetes complications due to prediabetes or poor management of diabetes was estimated using the population attribution fraction (PAF) approach.^24^ For each complication *c*, we calculated the indirect health expenditure attributable to prediabetes or uncontrolled glycemic status (DALY_c_) in 2002, 2009, and 2017 with the following formula by multiplying the PAF_*i*_, number of patients with diabetes complications (N_*i*_), and complication‐specific rate of DALYs (D_*i*_, years/100000 person) in each age or sex subgroup and then sum them up.

$${\text{DALY}}_{c}=\sum_{i}{\mathrm{PAF}}_{i}\times N_{i}\times D_{i}$$

The age‐ and sex‐specific parameters for estimations of DALYs are presented in Table S1. Using Levin's formula, PAF_*i*_ for prediabetes in subgroup *i* was based on the prevalence of prediabetes ($P_{i}$) in the surveys and the risk ratio (${\mathrm{RR}}_{i}$) of diabetes complications for prediabetes relevant to normal subjects.^25^, ^26^ ${\mathrm{PAF}}_{i}$ for diabetes in subgroup *i* was based on the weighted high FPG rate ($P_{i}$) in diagnosed and undiagnosed diabetes patients and the risk ratio (${\mathrm{RR}}_{i}$) for high versus low FPG.^27^

$${\mathrm{PAF}}_{i}=\frac{P_{i}\times \left({\mathrm{RR}}_{i}-1\right)}{P_{i}\times \left({\mathrm{RR}}_{i}-1\right)+1}\times 100\%$$

For prediabetes, the number of diabetes complications ($N_{i}$) were calculated as the product of prevalence of prediabetes (derived from current study), incidence of cardiovascular disease (CVD) and diabetic nephropathy (DN) in prediabetes (extracted from previous studies^26^, ^28^) and the number of the population in Shanghai (obtained from the National Bureau of Statistics) (https://data.stats.gov.cn/). Diabetes complications for high FPG ($N_{i}$) were estimated as the product of rate of high FPG, number of adult populations in Shanghai, and the incidence of ischemic heart disease (IHD), stroke, or DN in high FPG.^29^ Sex‐ and age‐specific DALYs ($D_{i}$) of IHD, stroke, and DN in China were derived from Global Health Data Exchange (http://ghdx.healthdata.org/) (Table S1).

### 2.6 Statistical analysis

All analyses were performed by sex using SAS 9.4 (SAS Institute, Cary, NC, USA). Data were presented as median (interquartile range) for continuous variables or as count (percentage) for categorical variables. Crude prevalence and 95% confidence interval (CI) of diabetes and prediabetes were calculated overall and by sex, age group, or birth cohort (ie, subjects born in certain similar calendar years) in each survey. Cochran‐Armitage trend test was used to examine the trends in prevalence, awareness, and glycemic control rates of diabetes over the years. Age‐standardized prevalence were estimated using the direct method based on the China national population data released by National Bureau of Statistics in 2019 (http://data.stats.gov.cn/easyquery.htm?cn=C01). Average annual change (AAC) in prevalence was calculated by dividing difference in prevalence by number of years over a similar period. Generalized linear model was used to compare the levels of FPG, 2hPG, and HbA1c among normal participants. Sensitivity analysis was performed by excluding subjects who participated in the 2009 survey from the 2017 survey.

All tests were two sided, and *p* values <.05 were considered statistically significant. Bonferroni correction was further applied to multiple comparisons.

## 3 RESULTS

### 3.1 Characteristics of the study participants

The participants of the three surveys differed significantly in age, educational level, drinking, smoking, and family history of diabetes (all *p* values <.01). The average levels of BMI, WC, FPG, 2hPG, and HbA1c were also observed to increase along with the three surveys in both men and women (*p* for trend <.01) (Table 1).

**TABLE 1:** Demographic and lifestyle characteristics of the participants of the surveys conducted in 2002–2003, 2009, and 2017.

	Men			*p* values	Women			*p* values
	The 2002–2003 survey (*n* = 5023)	The 2009 survey (*n* = 3456)	The 2017 survey (*n* = 7588)		The 2002–03 survey (*n* = 7279)	The 2009 survey (*n* = 3958)	The 2017 survey (*n* = 11 372)	
Age (years)	54.0 (46.0, 64.0)	55.0 (48.0, 61.0)	63.0 (56.0, 68.0)	***<.01***	51.0 (45.0, 61.0)	55.0 (49.0, 61.0)	62.0 (55.0, 66.0)	***<.01***
Age groups (years)								
35–44	988 (19.7)	532 (15.4)	461 (6.1)	***<.01***	1615 (22.2)	538 (13.6)	624 (5.5)	***<.01***
45–54	1670 (33.3)	1181 (34.2)	1174 (15.5)		2800 (38.5)	1427 (36.1)	1917 (16.9)	
55–64	1135 (22.6)	1180 (34.1)	2900 (38.2)		1448 (19.9)	1401 (35.4)	4889 (43.0)	
65–74	1230 (24.5)	563 (16.3)	3053 (40.2)		1416 (19.5)	592 (15.0)	3942 (34.7)	
Educational level								
Primary school or below	1112 (22.3)	619 (17.9)	1346 (18.2)	***<.01***	2996 (41.4)	1077 (27.2)	2481 (23.4)	***<.01***
Junior high school	1768 (35.4)	1578 (45.7)	3672 (49.7)		2254 (31.1)	1785 (45.1)	4922 (46.4)	
Senior high school	1376 (27.6)	955 (27.6)	1678 (22.7)		1633 (22.6)	940 (23.8)	2551 (24.1)	
Junior college or above	739 (14.8)	303 (8.8)	700 (9.5)		357 (4.9)	154 (3.9)	649 (6.1)	
Drinking								
Ever	2034 (40.9)	1864 (54.0)	3878 (51.3)	***<.01***	171 (2.4)	199 (5.0)	379 (3.3)	***<.01***
Never	2937 (59.1)	1590 (46.0)	3688 (48.7)		7107 (97.7)	3758 (95.0)	10 983 (96.7)	
Smoking								
Ever	3089 (61.6)	2295 (66.5)	5174 (68.2)	***<.01***	127 (1.7)	75 (1.9)	127 (1.1)	***<.01***
Never	1925 (38.4)	1158 (33.5)	2409 (31.8)		7152 (98.3)	3879 (98.1)	11 236 (98.9)	
Family history of diabetes								
Yes	617 (12.5)	560 (16.4)	1479 (20.1)	***<.01***	955 (13.2)	742 (19.0)	2361 (21.3)	***<.01***
No	4328 (87.5)	2850 (83.6)	5868 (79.9)		6292 (86.8)	3167 (81.0)	8731 (78.7)	
WC (cm)	84.0 (77.0, 90.0)	85.0 (79.0, 91.0)	89.0 (83.0, 94.2)	***<.01***	78.0 (72.0, 84.0)	80.0 (74.0, 87.0)	83.0 (77.5, 89.0)	***<.01***
WC groups (cm)								
Central obesity^a^	1115 (22.3)	894 (25.9)	3178 (41.9)	***<.01***	2712 (37.4)	1924 (48.6)	6990 (61.5)	***<.01***
Normal	3887 (77.7)	2559 (74.1)	4410 (58.1)		4550 (62.7)	2033 (51.4)	4382 (38.5)	
BMI (kg/m^2^)	24.3 (22.1,26.3)	24.2 (22.2, 26.3)	25.1 (23.1, 27.2)	***<.01***	24.0 (21.9, 26.5)	24.0 (21.9, 26.4)	24.5 (22.5, 26.7)	***<.01***
BMI groups (kg/m^2^)								
Normal: <24.0	2320 (46.3)	1606 (46.5)	2675 (35.3)	***<.01***	3630 (49.9)	1992 (50.3)	4986 (43.8)	***<.01***
Overweight: 24.0 ~ 27.9	2110 (42.1)	1437 (41.6)	3595 (47.4)		2641 (36.3)	1417 (35.8)	4558 (40.1)	
Obesity: ≥28.0	584 (11.7)	410 (11.9)	1318 (17.4)		1005 (13.8)	548 (13.9)	1828 (16.1)	
FPG level (mmol/L)	5.0 (4.5, 5.6)	5.0 (4.6, 5.6)	5.7 (5.2, 6.7)	***<.01***	5.0 (4.5, 5.5)	5.0 (4.7, 5.5)	5.5 (5.2, 6.2)	***<.01***
2hPG level (mmol/L)	5.3 (4.3, 6.7)	6.0 (4.8, 7.4)	6.6 (5.1, 9.8)	***<.01***	5.5 (4.6, 6.6)	6.1 (5.1, 7.2)	6.6 (5.4, 8.9)	***<.01***
HbA1c level (%)	‐	5.6 (5.2, 6.0)	5.8 (5.5, 6.3)	***<.01***	‐	5.5 (5.2, 5.9)	5.8 (5.5, 6.2)	***<.01***

*Note*: Data presented as number (%) for categorical variables and as median (interquartile range) for continuous variables. *p* values for chi‐square tests or Kruskal–Wallis tests. Bold and italics indicate *p* value <.05.

Abbreviations: 2hPG. 2‐h postprandial blood glucose; BMI, body mass index; FPG, fasting plasma glucose; HbA1c, hemoglobin A1c; WC, waist circumference.

^a^ Defined as WC > 90 cm in men and > 80 cm in women according to the World Health Organization criteria.

### 3.2 Crude and standardized prevalence of diabetes and prediabetes

**FIGURE 1:**
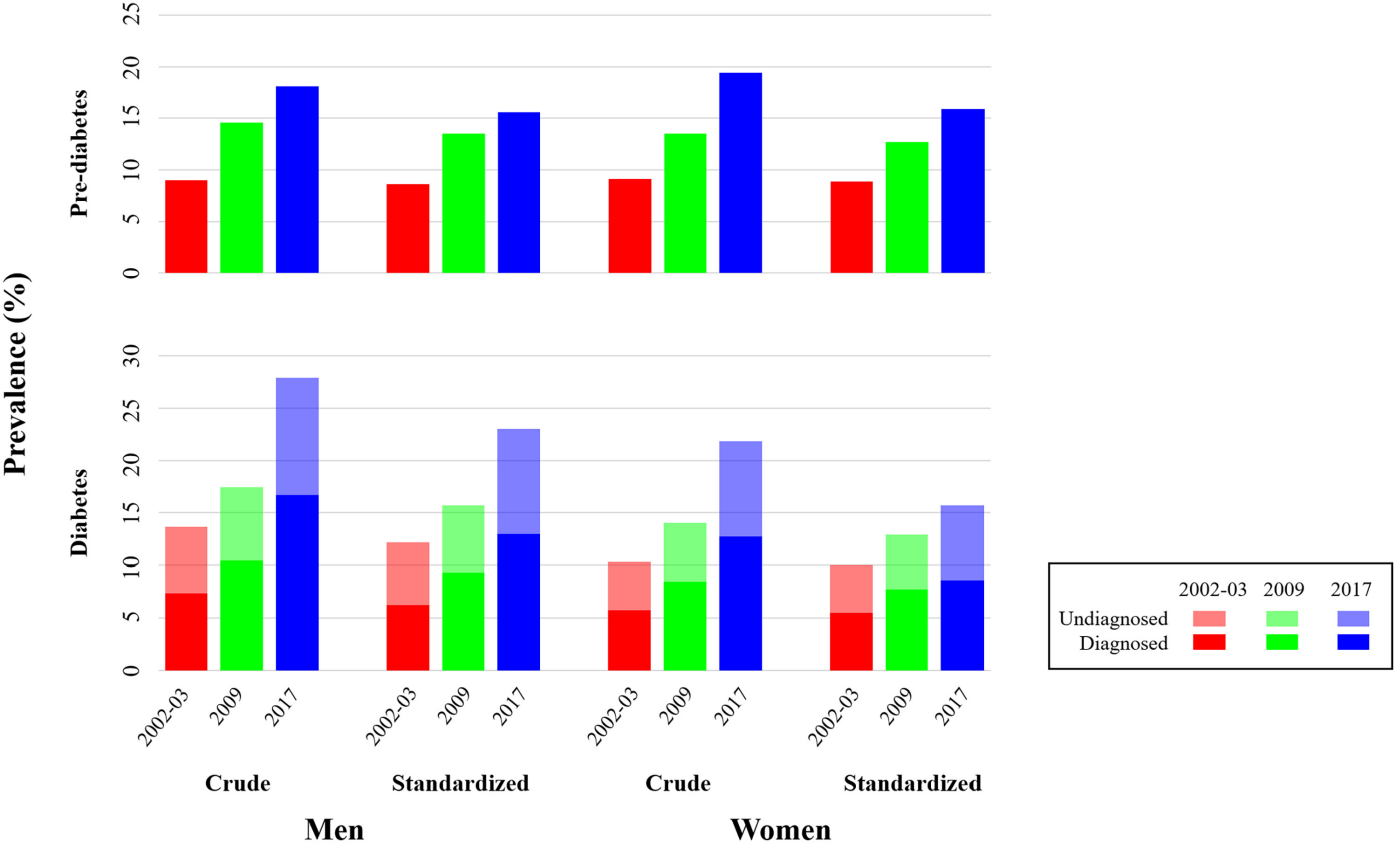
Prevalence of diabetes and prediabetes in Chinese men and women in 2002–2003, 2009, and 2017.

Figure 1 shows the increasing crude and standardized prevalence of diabetes (both diagnosed and undiagnosed) and prediabetes in participants of the three surveys. The crude prevalence and 95% CI of diabetes increased from 13.6% (12.7 ~ 14.6) in 2002–2003 to 17.5% (16.2 ~ 18.7) in 2009 and 27.9% (26.9 ~ 28.9) in 2017 in men, and from 10.3% (9.7 ~ 11.0) to 14.1% (13.0 ~ 15.2) and 21.8% (21.1 ~ 22.6), respectively, in women (all *p* for trend <.01). After adjusting for age, the prevalence and 95% CI were 12.2% (11.3 ~ 13.1), 15.7% (14.5 ~ 16.9), and 23.0% (22.1 ~ 24.0) in men in the three surveys and were 10.0% (9.3 ~ 10.7), 12.9% (11.8 ~ 13.9), and 15.7% (15.1 ~ 16.4%) in women (all *p* for trend <.01). The prevalence of diabetes increased more rapidly between 2009 and 2017 than between 2002 and 2003 to 2009 in men, with an AAC of 0.91% versus 0.50%, but not in women (0.35% vs. 0.41%) (Table 2).

**TABLE 2:** Trends and average annual changes in age‐adjusted prevalence of diabetes and prediabetes among Chinese men and women in Shanghai.

	Number of patients (%)			*p* for trend	AAC of prevalence (%)		
	The 2002–03 survey	The 2009 survey	The 2017 survey		2002 to 2009	2009 to 2017	2002 to 2017
**Diabetes**							
All subjects	1438 (11.1)	1160 (14.3)	4600 (19.4)	***<.01***	0.46	0.64	0.55
Men	685 (12.2)	603 (15.7)	2119 (23.0)	***<.01***	0.50	0.91	0.72
Educational level							
Primary school or below	139 (10.4)	83 (11.7)	334 (20.1)	***<.01***	0.19	1.05	0.65
Junior high school	241 (12.7)	284 (16.5)	1014 (22.2)	***<.01***	0.54	0.71	0.63
Senior high school	170 (12.2)	174 (16.7)	512 (24.4)	***<.01***	0.64	0.96	0.81
Junior college or above	129 (13.9)	61 (17.1)	201 (24.7)	***<.01***	0.46	0.95	0.72
*p* for trend	***.05***	***.02***	***<.01***				
BMI groups (kg/m^2^)							
< 24.0	207 (8.3)	225 (12.8)	592 (16.9)	***<.01***	0.65	0.51	0.58
24.0 ~ 27.9	352 (14.6)	266 (16.7)	1055 (23.8)	***<.01***	0.30	0.89	0.62
≥ 28.0	120 (17.7)	110 (24.0)	472 (31.1)	***<.01***	0.89	0.89	0.89
*p* for trend	***<.01***	***<.01***	***<.01***				
WC groups (cm)							
≤ 90	440 (10.3)	369 (13.2)	1031 (18.9)	***<.01***	0.41	0.71	0.57
> 90	239 (18.1)	232 (23.0)	1088 (28.6)	***<.01***	0.69	0.70	0.70
Women	753 (10.0)	557 (12.9)	2481 (15.7)	***<.01***	0.41	0.35	0.38
Educational level							
Primary school or below	402 (10.4)	209 (14.4)	606 (16.0)	***<.01***	0.57	0.20	0.37
Junior high school	197 (10.4)	228 (12.7)	1020 (15.5)	***<.01***	0.33	0.35	0.34
Senior high school	115 (9.0)	103 (11.9)	564 (17.4)	***<.01***	0.41	0.69	0.56
Junior college or above	31 (9.1)	17 (8.4)	101 (11.7)	.15	−0.10	0.41	0.17
*p* for trend	*.13*	***.02***	*.58*				
BMI groups (kg/m^2^)							
< 24.0	258 (7.6)	192 (9.5)	828 (11.6)	***<.01***	0.27	0.26	0.27
24.0 ~ 27.9	305 (10.6)	227 (13.7)	1061 (16.7)	***<.01***	0.45	0.36	0.40
≥ 28.0	190 (17.3)	138 (22.6)	592 (26.4)	***<.01***	0.76	0.48	0.61
*p* for trend	***<.01***	***<.01***	***<.01***				
WC groups (cm)							
≤ 80	270 (6.4)	179 (9.2)	669 (11.1)	***<.01***	0.39	0.24	0.31
> 80	479 (15.1)	378 (16.9)	1812 (19.4)	***<.01***	0.27	0.31	0.29
**Prediabetes**							
All subjects	1118 (8.7)	1039 (13.1)	3575 (15.8)	***<.01***	0.63	0.34	0.47
Men	454 (8.6)	503 (13.5)	1372 (15.6)	***<.01***	0.70	0.26	0.47
Educational level							
Primary school or below	118 (9.8)	84 (12.5)	270 (26.5)	***<.01***	0.39	1.75	1.11
Junior high school	157 (8.6)	229 (13.9)	672 (16.1)	***<.01***	0.76	0.28	0.50
Senior high school	118 (8.6)	141 (14.5)	291 (14.3)	***<.01***	0.84	−0.02	0.38
Junior college or above	60 (7.7)	49 (12.5)	104 (14.5)	***<.01***	0.69	0.25	0.45
*p* for trend	.15	.70	***<.01***				
BMI groups (kg/m^2^)							
< 24.0	152 (6.1)	186 (11.0)	394 (12.9)	***<.01***	0.70	0.23	0.45
24.0 ~ 27.9	218 (9.8)	221 (13.9)	682 (15.2)	***<.01***	0.58	0.16	0.36
≥ 28.0	84 (14.1)	96 (21.9)	296 (21.1)	***<.01***	1.11	−0.11	0.46
*p* for trend	***<.01***	***<.01***	***<.01***				
WC groups (cm)							
≤ 90	296 (7.3)	318 (11.7)	712 (13.3)	***<.01***	0.64	0.19	0.40
> 90	156 (13.4)	185 (18.8)	660 (19.0)	***<.01***	0.76	0.03	0.37
Women	664 (8.9)	536 (12.7)	2203 (15.9)	***<.01***	0.54	0.40	0.47
Educational level							
Primary school or below	366 (11.1)	175 (15.6)	544 (14.8)	***<.01***	0.64	−0.10	0.25
Junior high school	169 (7.9)	220 (12.7)	966 (16.9)	***<.01***	0.69	0.53	0.60
Senior high school	103 (7.5)	116 (12.9)	439 (13.7)	***<.01***	0.77	0.10	0.41
Junior college or above	20 (6.1)	25 (20.1)	85 (13.2)	***<.01***	2.00	−0.86	0.47
*p* for trend	***<.01***	.62	.05				
BMI groups (kg/m^2^)							
< 24.0	219 (6.3)	191 (9.5)	777 (12.4)	***<.01***	0.47	0.36	0.41
24.0 ~ 27.9	292 (10.6)	230 (15.0)	960 (17.7)	***<.01***	0.63	0.34	0.47
≥ 28.0	150 (13.9)	114 (19.0)	466 (22.9)	***<.01***	0.73	0.49	0.60
*p* for trend	***<.01***	***<.01***	***<.01***				
WC groups (cm)							
≤ 80	291 (6.5)	198 (10.0)	701 (13.2)	***<.01***	0.50	0.39	0.44
> 80	370 (12.8)	337 (16.3)	1502 (18.3)	***<.01***	0.50	0.26	0.37

*Note*: Prevalence adjusted for age and sex for all subjects and adjusted for age in stratified analysis by sex. Bold and italics indicate *p* value <.05.

Abbreviations: AAC, average annual change; BMI, body mass index; WC, waist circumference.

The increasing trends were also observed for crude and age‐adjusted prevalence of prediabetes in both men and women over the period (all *p* for trend <.01). Adjusting for age lowered the prevalence (95% CI) from 9.0% (8.3 ~ 9.8%), 14.6% (13.4 ~ 15.7%), and 18.1% (17.2 ~ 19.0%) to 8.6% (7.8 ~ 9.3%), 13.5% (12.4 ~ 14.7%), and 15.6% (14.8 ~ 16.5%) in men and from 9.1% (8.5 ~ 9.8%), 13.5% (12.5 ~ 14.6%), and 19.4% (18.7 ~ 20.1%) to 8.9% (8.2 ~ 9.5%), 12.7% (11.7 ~ 13.7%), and 15.9% (15.3 ~ 16.6%) in women. The prevalence of prediabetes increased slower between 2009 and 2017 than between 2002 and 2003 and 2009 in both men (AAC: 0.26% vs. 0.70%) and women (AAC: 0.40% vs. 0.54%) (Table 2).

### 3.3 Prevalence trends of diabetes and prediabetes by age, birth year, educational level, BMI, and WC

**FIGURE 2:**
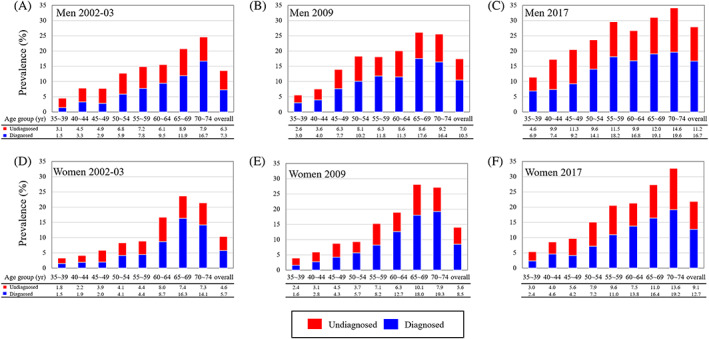
Age‐specific prevalence of diagnosed and undiagnosed diabetes among Chinese men and women in 2002–2003, 2009, and 2017. (A) Men in 2003‐03. (B) Men in 2009. (C) Men in 2017. (D) Women in 2002‐03. (E) Women in 2009 (F) Women in 2017.

**FIGURE 3:**
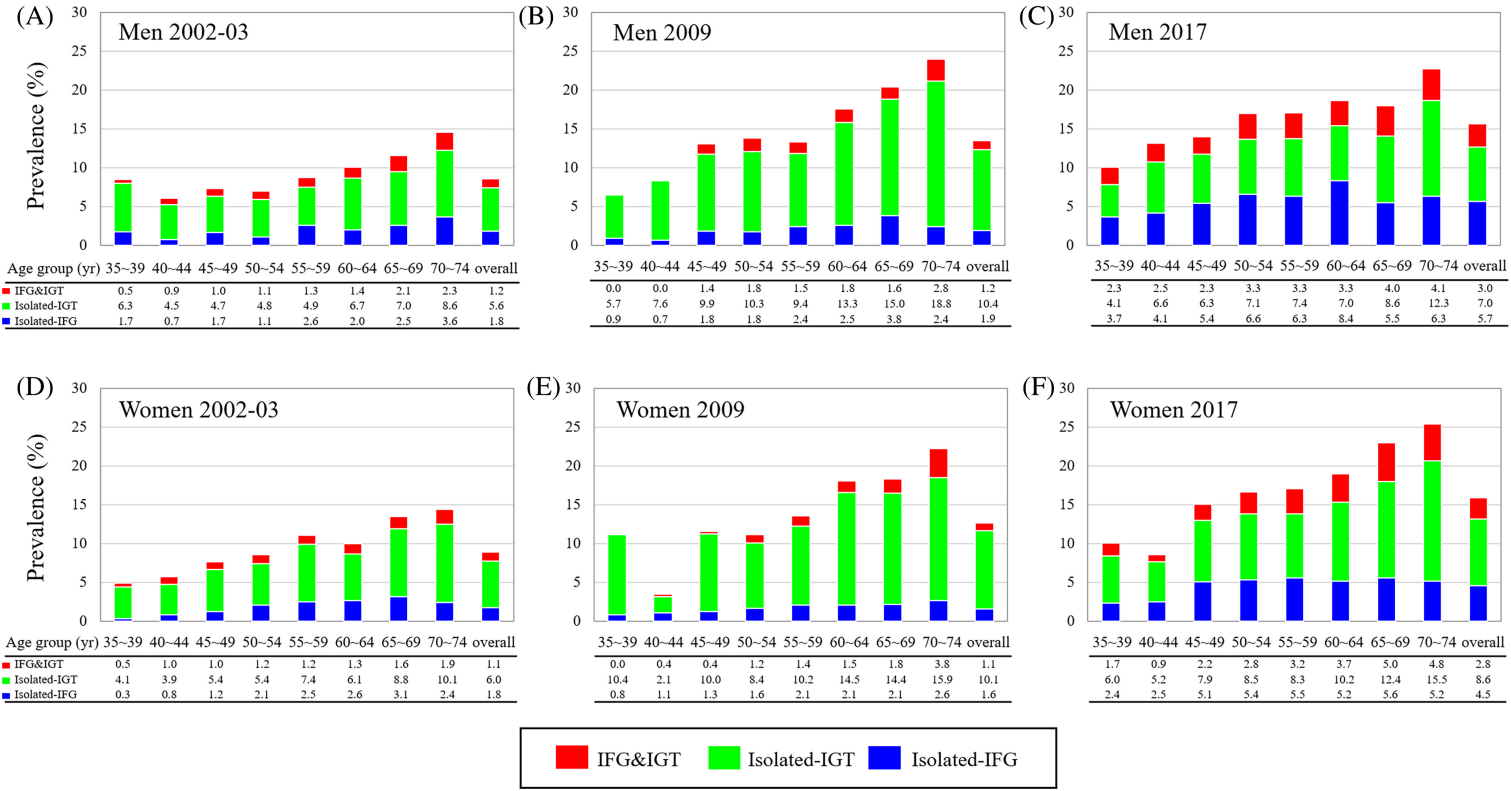
Age‐specific prevalence of prediabetes among Chinese men and women in 2002–2003, 2009, and 2017. IFG, impaired fasting glucose; IGT, impaired glucose tolerance. (A) Men in 2002‐02, (B) Men in 2009. (C) Men in 2017. (D) Women in 2002‐03. (E) Women in 2009. (F) Women in 2017.

Generally, the prevalence of diabetes increased with age in both men and women in each survey (*p* for trend <.05). Awareness rates of diabetes also increased with age in each survey and over the three surveys. As presented in Figure 2, awareness rates of diabetes were 31.6 ~ 60.0% in men and 40.0 ~ 45.0% in women at age of 35–39 years in the three surveys and reached 57.4 ~ 67.9% in men and 58.5 ~ 70.8% in women at age of 70–74 years. As a result, the overall awareness rates increased from 53.7% to 60.0% and 59.8% in men over the three surveys and from 55.5% to 60.3% and 58.2% in women. The prevalence of prediabetes increased with age but slightly fluctuated across several age groups. The age‐specific and age‐adjusted prevalence of isolated IGT appeared higher in participants of the 2009 survey, whereas those of isolated‐IFG and IGT and IFG were higher in 2017 survey among both men and women (Figure 3).

Figure S2 presents the prevalence of diabetes and prediabetes in the three surveys by birth cohort at each age group. An upward trend was observed for each birth cohort across the surveys, but the differences across the birth cohorts at same age group were not significant. Further stratified analysis by educational level showed increasing prevalence of diabetes along with educational level in men but a slightly decreasing trend in women (Table 2). The prevalence of prediabetes, on the other hand, was higher in subjects with lower educational levels, but the decreasing trend was significant only among men in the 2017 survey and among women in the 2002–2003 survey. The prevalence of diabetes and prediabetes increased with BMI in all the three surveys (*p* for trend <.01) and were higher among subjects with central obesity (*p* < .01).

### 3.4 Adjusted average levels of FPG, 2hPG, and HbA1c in participants with normal glucose

The average levels of FPG and 2hPG in normal participants of the three surveys were presented in Table S2. After adjusting for age and sex, FPG level was significantly higher in the 2017 survey than in the 2002–2003 and the 2009 surveys (*p* < .01), whereas 2hPG level was significantly higher in the 2009 survey than in the 2002–2003 and the 2017 surveys (*p* < .01). The average level of HbA1c was also higher in normal participants of the 2017 survey than those of the 2009 survey. Levels of FPG, 2hPG, and HbA1c all significantly increased with age among men and women in three surveys (all *p* values for trend <.01).

### 3.5 Glycemic control rates in diagnosed diabetes patients

When using FPG as an index, the glycemic control rate in diagnosed diabetes patients was higher in the 2009 survey but lower in the 2017 survey than the 2002–2003 survey. Further stratified analyses showed an upward trend of control rate along with age and educational level, particularly in the 2017 survey. Although the proportion of patients with FPG at 7.0–8.0 mmol/L remained stable over the three surveys, those with FPG >8.0 mmol/L accounted for a higher portion (55.4% for men, 47.3% for women) in 2017 (Table S3). When using HbA1c as an index, glycemic control rate was significantly lower in the 2017 survey than the 2009 survey, and the decreases were observed in all subgroups stratified by age, educational level, BMI, and WC (Table S4).

### 3.6 Estimated DALYs due to prediabetes and uncontrolled diabetes indicated by FPG level

Table 3 shows the estimated PAF and DALYs by diabetes complications due to prediabetes and uncontrolled diabetes. The PAF and DALY rates increased with increasing prevalence of prediabetes and high FPG rate. The total DALYs due to prediabetes was high in 2009 (3.94 and 417.51 DALYs for DN and CVD) and 2017 (5.95 and 528.73 DALYs for DN and CVD) tripled those of 2002 (1.09 and 126.87 DALYs for DN and CVD), whereas the DALYs due to uncontrolled FPG was the highest in 2017 (37.22, 375.29, and 363.14 DALYs for DN, IHD, and stroke).

**TABLE 3:** Estimated disability adjusted life years of diabetes complications due to prediabetes and uncontrolled diabetes in Shanghai in 2002, 2009, and 2017.

	Men			Women			Total		
	2002	2009	2017	2002	2009	2017	2002	2009	2017
**Uncontrolled diabetes**									
High FPG (%)^a^ ^,^ ^b^	10.09	11.46	20.26	8.15	8.93	13.29	9.12	10.20	16.80
PAF (%)^b^									
Diabetic nephropathy	3.74	4.23	7.25	3.03	3.32	4.85	3.39	3.78	6.06
Cardiovascular disease									
Ischemic heart disease	1.94	2.20	3.89	1.53	1.69	2.51	1.74	1.95	3.20
Stroke	1.95	2.21	3.89	1.55	1.71	2.53	1.75	1.96	3.22
DALY rate (per 100 000)^b^ ^,^ ^c^									
Diabetic nephropathy	8.82	9.73	15.77	7.86	7.99	11.37	8.34	8.86	13.58
Cardiovascular disease									
Ischemic heart disease	83.03	96.80	157.15	47.10	47.83	61.58	65.17	72.45	109.63
Stroke	169.49	149.94	206.83	107.13	83.51	94.28	138.49	116.91	150.87
Number of complications (thousand)									
Diabetic nephropathy	35.88	61.05	129.41	29.24	48.69	83.39	65.12	109.74	212.79
Cardiovascular disease									
Ischemic heart disease	44.06	74.97	158.90	35.91	59.78	102.39	79.96	134.75	261.30
Stroke	30.69	52.23	110.70	25.02	41.65	71.34	55.71	93.88	182.04
DALYs									
Diabetic nephropathy	4.03	7.13	23.57	3.52	5.32	13.65	7.55	12.45	37.22
Cardiovascular disease									
Ischemic heart disease	46.24	85.80	282.35	26.84	39.63	92.93	73.08	125.43	375.29
Stroke	68.01	93.84	263.90	42.85	48.23	99.24	110.85	142.08	363.14
**Prediabetes**									
Prevalence of prediabetes (%)^b^	8.56	13.51	15.64	8.88	12.71	15.94	8.72	13.11	15.79
PAF (%)^b^									
Diabetic nephropathy	3.70	5.69	6.56	3.83	5.37	6.66	3.76	5.53	6.61
Cardiovascular disease	0.85	1.33	1.54	0.88	1.25	1.57	0.86	1.29	1.55
DALY rate (per 100 000)^b^ ^,^ ^c^									
Diabetic nephropathy	7.94	13.25	13.90	8.62	11.77	14.02	8.28	12.52	13.96
Cardiovascular disease	114.96	174.48	163.74	86.36	103.42	103.02	100.74	139.15	133.55
Number of complications (thousand)									
Diabetic nephropathy	5.80	13.05	17.97	5.98	12.71	18.12	11.78	25.76	36.08
Cardiovascular disease	53.91	121.32	166.96	55.54	118.07	168.34	109.45	239.38	335.30
DALYs									
Diabetic nephropathy	0.50	2.14	2.81	0.59	1.80	3.14	1.09	3.94	5.95
Cardiovascular disease	69.77	265.81	308.28	57.09	151.70	220.44	126.87	417.51	528.73

Abbreviations: DALYs, disability‐adjusted life‐years; FPG, fasting plasma glucose; PAF, population attribution fraction.

^a^ Calculated as prevalence of diagnosed diabetes×uncontrolled rate + prevalence of undiagnosed diabetes;

^b^ Weighted by age;

^c^ Calculated as PAF × DALY rates of all risks.

### 3.7 Sensitivity analyses

Excluding 1783 subjects who participated in both the 2009 and the 2017 surveys did not change the results substantially. In sensitivity analysis, the age‐standardized prevalence (95% CI) of diabetes and prediabetes were 19.7% (19.1 ~ 20.3) and 15.8% (15.2 ~ 16.3) in 2017, respectively, very close to 19.4% (18.8 ~ 20.0) and 15.8% (15.3 ~ 16.3) in the main analysis, with all *p* values for differences >.05.

## 4 DISCUSSION

This study describes the levels and trends of prevalence and management of diabetes and prediabetes over past years based on the randomly‐selected samples of adults in Shanghai, China. The main findings included (a) the prevalence of diabetes in the population almost doubled over the 15‐year period, and was as high as more than 20% in 2017; (b) the prevalence of prediabetes also increased over the three surveys; (c) the improved awareness of diabetes over the 15‐year period was along with the decreasing glycemic control rates among diagnosed patients; (d) average levels of FPG, 2hPG, and HbA1c increased in normal subjects over the years; and (e) the changes in prevalence and control rate of diabetes may have contributed to an increasing burden of diabetes complications.

The trends in prevalence of diabetes in China have been investigated extensively.^10^, ^30^, ^31^ In this study, both crude and age‐adjusted prevalence of diabetes in Shanghai was at a higher level than the average levels in China during same period and increased rapidly over the 15‐year survey period. The AAC in our subjects (1.0% in men and 0.8% in women) was also slightly higher than those in other areas of China,^32^ Korea,^33^ and the United States.^34^ Several factors may explain the upward trends in prevalence. First, the widening crude‐adjusted prevalence gap over the three surveys suggests more contribution of population aging in the epidemic of diabetes, a disorder related with aging.^35^ More important, the upward trend of age‐adjusted prevalence of diabetes indicates elevated risk exposures. Rapid urbanization in China in recent decades has led to the adoption of western lifestyle characterized with more high‐energy diet intake but less physical activities.^36^ The sedentary lifestyle is particularly popular in middle‐aged people, leading to more rapid increase of diabetes prevalence in the subpopulation.^37^, ^38^, ^39^, ^40^ Moreover, Eastern Asians have lower compensatory β‐cell function than people from Europe and Africa,^41^ which may interact with elevated risk exposures and lead to excess risk of diabetes. Interestingly, the prevalence of diabetes increased with educational level in men but showed a slightly decreased trend in women, demonstrating sex heterogeneous association pattern and indicating sex‐specific risk factors for diabetes.

Prediabetes refers to an intermediary hyperglycemia stage between normal glucose status and overt diabetes,^42^, ^43^, ^44^, ^45^ which confers a higher risk of diabetes. It has been suggested that prediabetes patients who progress to diabetes may represent a particularly high‐risk group for major adverse cardiac events.^46^ In this study, the age‐adjusted prevalence of prediabetes also increased over the three surveys, with isolated IFG increasing and isolated IGT decreasing from 2009 to 2017. The trends were also reported by an Indian study in which the pools of individuals with IGT began to shrink while diabetes populations were expanding.^47^ IFG and IGT are heterogeneous pathophysiologic disorders,^48^ which differ in progression to overt diabetes.^49^ The notable elevated levels of FPG and 2hPG in normal subjects over the three surveys also indicate that the population were more susceptible to prediabetes and type 2 diabetes. These findings may imply a more rapid transition from normoglycemic to prediabetes and from prediabetes to diabetes in Chinese populations.

An intriguing observation in this study is the increasing awareness of diabetes over the years. The positive change may be due to the effort of Chinese government to curb the epidemic of the disease. In 2012, China government issued the National Plan for Non‐Communicable Disease Prevention and Treatment, proposing multidimensional solutions to address the heavy burden of diabetes and other common noncommunicable diseases.^50^ Screening of diabetes and prediabetes was conducted more frequently and became one of primary healthcare services for the elderly, which may account for the overall improved awareness and the higher level in the elderly.

Along with the increasing awareness of diabetes was the decreasing glycemic control rates in physician‐diagnosed patients. About half of diagnosed patients had FPG level higher than 8.0 mmol/L and about one‐third diagnosed patients had HbA1c level higher than 8.0% (64 mmol/mol). The adverse changes in control rates coincided with the transferred management of diabetes from tertiary hospitals to CHCs, indicating the potential contribution of the big gap between general practitioners and endocrinologists in clinic abilities and the trusts from patients.^51^ We also found that the glycemic control rate was lower in younger or less educated patients, which may be attributed to popular sedentary lifestyles and unwillingness to visit hospitals in the populations.^52^, ^53^

The declined glycemic control rate among diagnosed patients and the increasing prevalence of diabetes and prediabetes have collectively resulted in heavy burden of diabetes‐related complications, with the largest increase in DALYs for CVDs and DN. These results indicate that the management of diabetes should improve glycemic control status of diabetes patients, but also attempt to lower prevalence of diabetes and prediabetes.

There are several strengths in this study. First, the three cross‐sectional studies followed a similar multistage stratified sampling procedure, maximizing the representation of the subjects. Second, the vigorous personnel training, standardized data collection procedure and laboratory assays guarantee the quality of data. Furthermore, the trend analyses were conducted in subgroups classified by sex, age group, birth cohort, and educational level, allowing us to describe the trends of prevalence, awareness, and control rates comprehensively. Finally, the extensive interpopulation comparisons of prevalence and trends of diabetes make it possible to estimate the disease burden of diabetes complications.

Limitations in this study also should be mentioned. First, the three surveys were incomparable in sampling framework, sample size, and response rates to investigation, which may have induced selection bias to the results. Second, we could not exclude the possible bias of batch in questionnaire‐based surveys, body measurements, and biochemistry assays, even though a similar standardized protocol was followed in the three surveys. Moreover, lack of data on HbA1c in 2002–2003 limited our ability to describe the trend of HbA1c goal achievement rates over the three surveys. Instead, we had to use FPG as an index. Finally, due to the cross‐sectional design, we could not follow‐up the subjects for incidence of complications and were unable to directly estimate and project‐related disease burdens.

## 5 CONCLUSION

The current study demonstrated that a large proportion of Chinese adults are suffering from type 2 diabetes and prediabetes. Despite the improved awareness, the increasing prevalence of diabetes and prediabetes and decreasing glycemic control rates in diagnosed patients may contribute to an increasing burden of diabetes complications in the populations. Our results highlight the necessary to further strengthen the healthcare system in China to guarantee extensive management of diabetes patients in CHCs.

## DISCLOSURE

The authors declare that they have no known competing financial interests or personal relationships that could have appeared to influence the work reported in this paper.

## Supporting information


**TABLE S1.** Age‐ and sex specific parameters used to calculate disability adjusted life years (DALYs) of diabetes complications due to prediabetes and uncontrolled diabetes
**TABLE S2.** Levels of fasting plasma glucose (FPG), 2‐h postprandial blood glucose (2hPG), and hemoglobin A1c (HbA1c) in normal Chinese men and women in 2002–2003, 2009, and 2017.
**TABLE S3.** Glycemic control rates by fasting plasma glucose (FPG) level among diagnosed diabetes patients
**TABLE S4.** Glycemic control rates by hemoglobin A1c (HbA1c) level among diagnosed diabetes patients
**FIGURE S1.** Flow chart of participant recruitment in the 2002–2003 (A), 2009 (B), and 2017 (C) survey.
**FIGURE S2.** Prevalence of diabetes and prediabetes by birth year and age group among Chinese men and women in 2002–2003, 2009, and 2017.
