# Supplementary material for: Prevalence trends and disease burden of diabetes and prediabetes in Chinese adults of Shanghai
Source: J Diabetes. 2023 May 18;15(7):583–96. doi: 10.1111/1753-0407.13391 (PMC10345979; doi:10.1111/1753-0407.13391)
Supplement: Supplementary file 1 — TABLE S1. Age‐ and sex specific parameters used to calculate disability adjusted life years (DALYs) of diabetes complications due to prediabetes and uncontrolled diabetes TABLE S2. Levels of fasting plasma glucose (FPG), 2‐h postprandial blood glucose (2hPG), and hemoglobin A1c (HbA1c) in normal Chinese men and women in 2002–2003, 2009, and 2017. TABLE S3. Glycemic control rates by fasting plasma glucose (FPG) level among diagnosed diabetes patients TABLE S4. Glycemic control rates by hemoglobin A1c (HbA1c) level among diagnosed diabetes patients FIGURE S1. Flow chart of participant recruitment in the 2002–2003 (A), 2009 (B), and 2017 (C) survey. FIGURE S2. Prevalence of diabetes and prediabetes by birth year and age group among Chinese men and women in 2002–2003, 2009, and 2017. [file JDB-15-583-s001.docx]

**Supplementary Table 1** Age and sex specific parameters used to calculate DALYs of diabetes complications due to prediabetes and uncontrolled diabetes

|  | Men | | |  | Women | | |
| --- | --- | --- | --- | --- | --- | --- | --- |
|  | 2002-2003 | 2009 | 2017 |  | 2002-2003 | 2009 | 2017 |
| Prevalence of diagnosed diabetes (%) | | |  |  |  |  |  |
| 35~39 | 1.45 | 3.04 | 6.88 |  | 1.46 | 1.59 | 2.35 |
| 40-44 | 3.31 | 3.97 | 7.41 |  | 1.9 | 2.79 | 4.6 |
| 45~49 | 2.87 | 7.67 | 9.23 |  | 1.95 | 4.28 | 4.17 |
| 50-54 | 5.88 | 10.16 | 14.11 |  | 4.13 | 5.66 | 7.17 |
| 55~59 | 7.8 | 11.83 | 18.17 |  | 4.42 | 8.21 | 10.96 |
| 60-64 | 9.47 | 11.52 | 16.81 |  | 8.69 | 12.65 | 13.78 |
| 65~69 | 11.87 | 17.57 | 19.05 |  | 16.27 | 18.04 | 16.36 |
| 70-74 | 16.73 | 16.4 | 19.61 |  | 14.1 | 19.25 | 19.16 |
| Uncontrol rate of diagnosed diabetes patients (%) | | | | | |  |  |
| 35~39 | 100.00 | 42.86 | 86.67 |  | 77.78 | 50.00 | 85.71 |
| 40-44 | 68.42 | 50.00 | 77.78 |  | 57.89 | 25.00 | 73.33 |
| 45~49 | 54.17 | 58.82 | 85.37 |  | 75.86 | 62.50 | 88.46 |
| 50-54 | 75.51 | 64.00 | 77.67 |  | 74.07 | 55.10 | 74.73 |
| 55~59 | 63.27 | 58.23 | 78.45 |  | 59.46 | 62.69 | 68.87 |
| 60-64 | 70.83 | 52.54 | 79.38 |  | 64.15 | 52.70 | 68.55 |
| 65~69 | 61.25 | 54.55 | 75.49 |  | 66.92 | 37.29 | 66.34 |
| 70-74 | 62.37 | 36.59 | 72.05 |  | 64.37 | 41.18 | 66.90 |
| Prevalence of undiagnosed diabetes (%) | | | |  |  |  |  |
| 35~39 | 3.14 | 2.61 | 4.59 |  | 1.79 | 2.39 | 3.02 |
| 40-44 | 4.53 | 3.64 | 9.88 |  | 2.20 | 3.14 | 3.99 |
| 45~49 | 4.90 | 6.32 | 11.26 |  | 3.89 | 4.46 | 5.56 |
| 50-54 | 6.84 | 8.13 | 9.59 |  | 4.13 | 3.70 | 7.88 |
| 55~59 | 7.17 | 6.29 | 11.45 |  | 4.42 | 7.11 | 9.61 |
| 60-64 | 6.11 | 8.59 | 9.87 |  | 8.03 | 6.32 | 7.48 |
| 65~69 | 8.90 | 8.63 | 12.04 |  | 7.38 | 10.09 | 10.97 |
| 70-74 | 7.91 | 9.20 | 14.55 |  | 7.29 | 7.92 | 13.59 |
| Prevalence of prediabetes (%) | | |  |  |  |  |  |
| 35~39 | 8.45 | 6.52 | 10.09 |  | 4.88 | 11.16 | 10.07 |
| 40-44 | 6.10 | 8.28 | 13.17 |  | 5.70 | 3.48 | 8.59 |
| 45~49 | 7.29 | 13.09 | 13.96 |  | 7.65 | 11.59 | 15.12 |
| 50-54 | 6.96 | 13.82 | 16.99 |  | 8.56 | 11.20 | 16.71 |
| 55~59 | 8.76 | 13.32 | 17.07 |  | 11.10 | 13.60 | 17.05 |
| 60-64 | 10.06 | 17.58 | 18.70 |  | 10.00 | 18.12 | 19.03 |
| 65~69 | 11.57 | 20.45 | 18.04 |  | 13.52 | 18.35 | 23.00 |
| 70-74 | 14.57 | 24.00 | 22.77 |  | 14.42 | 22.26 | 25.48 |
| Population in Shanghai ^†^ | |  |  |  |  |  |  |
| 35~39 | 810702 | 976449 | 897781 |  | 865279 | 1036200 | 879870 |
| 40-44 | 611865 | 1037417 | 957413 |  | 631106 | 1094987 | 931307 |
| 45~49 | 625126 | 847714 | 1148575 |  | 656984 | 905524 | 1122669 |
| 50-54 | 537967 | 788175 | 1048424 |  | 572648 | 811985 | 1035200 |
| 55~59 | 384883 | 735266 | 647351 |  | 396087 | 766029 | 640885 |
| 60-64 | 305605 | 526453 | 728323 |  | 319991 | 529952 | 737042 |
| 65~69 | 274567 | 376566 | 541121 |  | 292603 | 384488 | 569211 |
| 70-74 | 207267 | 301929 | 337973 |  | 220824 | 314653 | 363982 |
| DALYs rate of ischemic heart disease (per100,000) ^‡^ | | | |  |  |  |  |
| 35~39 | 959.81 | 932.89 | 1045.77 |  | 444.86 | 325.61 | 286.70 |
| 40-44 | 1494.29 | 1562.04 | 1654.39 |  | 693.65 | 555.18 | 483.52 |
| 45~49 | 1932.15 | 2262.35 | 2175.61 |  | 1060.39 | 878.17 | 700.40 |
| 50-54 | 2962.20 | 3035.96 | 2999.57 |  | 1588.61 | 1257.48 | 1112.57 |
| 55~59 | 4053.25 | 4120.73 | 4119.29 |  | 2073.05 | 1962.94 | 1751.10 |
| 60-64 | 5276.15 | 6011.76 | 5619.85 |  | 3253.90 | 3348.89 | 2861.41 |
| 65~69 | 7448.81 | 7809.21 | 7548.82 |  | 4932.69 | 4951.19 | 4481.79 |
| 70-74 | 10672.86 | 11720.89 | 11194.75 |  | 7633.70 | 8213.78 | 7419.83 |
| DALYs rate of stroke (per100,000) ^‡^ | | |  |  |  |  |  |
| 35~39 | 1081.35 | 967.32 | 955.66 |  | 683.32 | 465.13 | 380.96 |
| 40-44 | 2082.74 | 1811.76 | 1587.77 |  | 1256.63 | 869.85 | 654.69 |
| 45~49 | 3000.97 | 2770.50 | 2254.27 |  | 2132.94 | 1437.72 | 1002.97 |
| 50-54 | 5128.01 | 4035.16 | 3396.77 |  | 3655.79 | 2290.38 | 1754.43 |
| 55~59 | 7639.59 | 5963.25 | 4995.73 |  | 4862.62 | 3610.70 | 2706.56 |
| 60-64 | 10906.81 | 9546.84 | 7562.88 |  | 7177.66 | 5771.15 | 4301.26 |
| 65~69 | 15948.08 | 12986.48 | 10919.46 |  | 10841.55 | 8465.65 | 6750.96 |
| 70-74 | 24157.37 | 19662.62 | 16664.08 |  | 16940.09 | 13116.16 | 10694.08 |
| DALYs rate of diabetic nephropathy (per100,000) ^‡^ | | | |  |  |  |  |
| 35~39 | 45.73 | 35.84 | 31.10 |  | 40.37 | 28.30 | 23.04 |
| 40-44 | 71.24 | 60.57 | 55.03 |  | 64.59 | 51.98 | 43.92 |
| 45~49 | 97.99 | 96.98 | 90.26 |  | 106.61 | 88.91 | 77.82 |
| 50-54 | 162.09 | 143.50 | 148.13 |  | 168.60 | 135.45 | 132.40 |
| 55~59 | 230.14 | 217.97 | 220.21 |  | 221.17 | 206.91 | 201.17 |
| 60-64 | 287.31 | 314.69 | 310.59 |  | 289.05 | 290.37 | 279.47 |
| 65~69 | 376.85 | 403.68 | 408.29 |  | 371.22 | 374.16 | 386.74 |
| 70-74 | 509.53 | 551.62 | 561.98 |  | 471.00 | 500.36 | 524.87 |
| DALYs rate of cardiovascular disease (per100,000) ^‡^ | | | |  |  |  |  |
| 35~39 | 2183.58 | 2031.63 | 2140.41 |  | 1233.48 | 861.40 | 723.22 |
| 40-44 | 3808.22 | 3599.12 | 3473.94 |  | 2120.86 | 1556.37 | 1244.34 |
| 45~49 | 5256.11 | 5363.31 | 4752.89 |  | 3476.08 | 2519.64 | 1866.75 |
| 50-54 | 8629.05 | 7544.44 | 6884.33 |  | 5721.81 | 3879.46 | 3155.30 |
| 55~59 | 12537.76 | 10792.70 | 9825.10 |  | 7618.04 | 6110.63 | 4920.21 |
| 60-64 | 17454.13 | 16715.18 | 14303.71 |  | 11509.96 | 10011.30 | 7934.72 |
| 65~69 | 25351.67 | 22491.18 | 20237.10 |  | 17514.23 | 14807.06 | 12557.90 |
| 70-74 | 38034.23 | 34063.78 | 30755.18 |  | 27464.10 | 23590.77 | 20402.70 |

^†^ Calculated with gender ratio, age weight, and total population in Shanghai derived from to the National Bureau of Statistics in 2022 (<https://data.stats.gov.cn/>)

^‡^ Derived from Global Health Data Exchange in 2022 (<http://ghdx.healthdata.org/>).

**Supplementary Table 2** Levels of FPG, 2hPG and HbA1c in normal Chinese men and women in 2002-03, 2009 and 2017.

|  | ls-mean (95%CI) of FPG (mmol/L) | | |  | ls-mean (95%CI) of 2hPG (mmol/L) | | |  | ls-mean (95%CI) of HbA1c (%) | |
| --- | --- | --- | --- | --- | --- | --- | --- | --- | --- | --- |
|  | 2002-03  (n= 9,746) | 2009  (n= 5,215) | 2017  (n= 10,785) |  | 2002-03  (n= 9,746) | 2009  (n= 5,215) | 2017  (n= 10,785) |  | 2009  (n= 5,215) | 2017  (n= 10,785) |
| **All subjects** | 4.85 (4.84, 4.86) | 4.89 (4.88, 4.91)^*^ | 5.26 (5.25, 5.27)^*#^ |  | 5.20 (5.18, 5.23) | 5.57 (5.54, 5.60)^*^ | 5.45 (5.43, 5.47)^*^ |  | 5.43 (5.42, 5.45) | 5.60 (5.59, 5.61)^#^ |
| **Men** | 4.85 (4.83, 4.87) | 4.86 (4.84, 4.88) | 5.28 (5.26, 5.30)^*#^ |  | 5.07 (5.03, 5.11) | 5.46 (5.41, 5.51)^*^ | 5.26 (5.22, 5.30)^*#^ |  | 5.43 (5.41, 5.44) | 5.58 (5.57, 5.60)^#^ |
| Age groups (years) |  |  |  |  |  |  |  |  |  |  |
| 35~44 | 4.68 (4.64, 4.72) | 4.69 (4.63, 4.74) | 5.19 (5.13, 5.25)^*#^ |  | 4.83 (4.75, 4.91) | 5.26 (5.15, 5.37)^*^ | 5.04 (4.91, 5.17)^*#^ |  | 5.25 (5.21, 5.29) | 5.43 (5.38, 5.48)^#^ |
| 45~54 | 4.83 (4.81, 4.86) | 4.83 (4.79, 4.87) | 5.28 (5.24, 5.32)^*#^ |  | 4.89 (4.83, 4.95) | 5.31 (5.22, 5.39)^*^ | 5.09 (5.00, 5.18)^*#^ |  | 5.43 (5.39, 5.47) | 5.53 (5.49, 5.57)^#^ |
| 55~64 | 4.87 (4.84, 4.90) | 4.93 (4.89, 4.96) | 5.31 (5.29, 5.34) ^*#^ |  | 5.11 (5.02, 5.19) | 5.51 (5.42, 5.60)^*^ | 5.29 (5.22, 5.35)^*#^ |  | 5.43 (5.40, 5.46) | 5.62 (5.60, 5.64)^#^ |
| 65~74 | 4.96 (4.93, 5.00) | 4.89 (4.83, 4.94) | 5.33 (5.31, 5.36)^*#^ |  | 5.37 (5.29, 5.46) | 5.72 (5.58, 5.86)^*^ | 5.55 (5.49, 5.61)^*^ |  | 5.46 (5.41, 5.50) | 5.64 (5.62, 5.66)^#^ |
| *P for trend ^†^* | *<0.001* | *<0.001* | *<0.001* |  | *<0.001* | *<0.001* | *<0.001* |  | *<0.001* | *<0.001* |
| **Women** | 4.86 (4.84, 4.87) | 4.92 (4.90, 4.94)^*^ | 5.26 (5.24, 5.27)^*#^ |  | 5.35 (5.32, 5.37) | 5.68 (5.64, 5.72)^*^ | 5.63 (5.60, 5.66)^*#^ |  | 5.44 (5.43, 5.46) | 5.61 (5.60, 5.62)^#^ |
| Age group (years) |  |  |  |  |  |  |  |  |  |  |
| 35~44 | 4.73 (4.70, 4.76) | 4.74 (4.69, 4.79) | 5.16 (5.12, 5.21)^*#^ |  | 5.12 (5.07, 5.17) | 5.39 (5.30, 5.48)^*^ | 5.24 (5.16, 5.33) |  | 5.25 (5.20, 5.29) | 5.39 (5.34, 5.43)^#^ |
| 45~54 | 4.85 (4.83, 4.88) | 4.91 (4.88, 4.95)^*^ | 5.22 (5.20, 5.25)^*#^ |  | 5.19 (5.14, 5.23) | 5.53 (5.46, 5.59)^*^ | 5.56 (5.50, 5.62)^*^ |  | 5.41 (5.38, 5.43) | 5.54 (5.53, 5.57)^#^ |
| 55~64 | 4.87 (4.84, 4.89) | 4.97 (4.94, 5.00)^*^ | 5.29 (5.27, 5.31)^*#^ |  | 5.45 (5.38, 5.51) | 5.80 (5.73, 5.87)^*^ | 5.69 (5.65, 5.73)^*^ |  | 5.48 (5.46, 5.51) | 5.66 (5.65, 5.67)^#^ |
| 65~74 | 4.94 (4.90, 4.97) | 4.95 (4.90, 5.01) | 5.34 (5.31, 5.36)^*#^ |  | 5.61 (5.54, 5.68) | 5.98 (5.86, 6.10)^*^ | 5.96 (5.91, 6.00)^*^ |  | 5.43 (5.39, 5.47) | 5.71 (5.69, 5.73)^#^ |
| *P for trend ^†^* | *<0.001* | *<0.001* | *<0.001* |  | *<0.001* | *<0.001* | *<0.001* |  | *<0.001* | *<0.001* |

Ls-mean adjusted for age and/or sex; ^*^ p<0.0167 compared with the 2002-03 survey by Bonferroni correction; ^#^ p<0.0167 compared with the 2009 survey by Bonferroni correction; *^†^ P* for trend using age as a continuous variable in generalized linear model.

FPG, fasting plasma glucose; 2hPG, 2-hour postprandial blood glucose; HbA1c, Hemoglobin A1c.

**Supplementary Table 3** Glycemic control rates by FPG level among diagnosed diabetes patients

|  | The 2003-03 survey (mmol/L) | | |  | The 2009 survey (mmol/L) | | |  | The 2017 survey (mmol/L) | | |
| --- | --- | --- | --- | --- | --- | --- | --- | --- | --- | --- | --- |
|  | <7.0 | 7.0~8.0 | >8.0 |  | <7.0 | 7.0~8.0 | >8.0 |  | <7.0 | 7.0~8.0 | >8.0 |
| **All subjects** | 266 (33.8) | 150 (19.1) | 370 (47.1) |  | 329 (47.1) ^*^ | 134 (19.2) | 235 (33.7) ^*^ |  | 746 (27.5) ^*^ | 579 (21.4) | 1,384 (51.1) ^*^ |
| **Men** | 127 (34.5) | 66 (17.9) | 175 (47.6) |  | 163 (45.0) ^*^ | 66 (18.2) | 133 (36.7) ^*^ |  | 292 (23.1) ^*^ | 273 (21.6) | 701 (55.4) ^*^ |
| Age group (years) |  |  |  |  |  |  |  |  |  |  |  |
| 35~44 | 6 (24.0) | 5 (20.0) | 14 (56.0) |  | 10 (52.6) | 2 (10.5) | 7 (36.8) |  | 6 (18.2) ^*^ | 4 (12.1) | 23 (69.7) |
| 45~54 | 23 (31.5) | 13 (17.8) | 37 (50.7) |  | 41 (37.6) | 19 (17.4) | 49 (45.0) |  | 29 (20.1) ^*^ | 34 (23.6) | 81 (56.3) |
| 55~64 | 32 (33.0) | 13 (13.4) | 52 (53.6) |  | 61 (44.2) | 27 (19.6) | 50 (36.2) ^*^ |  | 105 (21.0) ^*^ | 97 (19.4) | 299 (59.7) ^*^ |
| 65~74 | 66 (38.2) | 35 (20.2) | 72 (41.6) |  | 51 (53.1) | 18 (18.8) | 27 (28.1) |  | 152 (25.9) ^*^ | 138 (23.5) | 298 (50.7) ^*^ |
| *P for trend* | *0.12* | *0.67* | *0.07* |  | *0.12* | *0.50* | ***0.03*** |  | ***0.04*** | *0.20* | ***<0.01*** |
| Educational level |  |  |  |  |  |  |  |  |  |  |  |
| Primary school or below | 24 (36.4) | 8 (12.1) | 34 (51.5) |  | 19 (38.0) | 8 (16.0) | 23 (46.0) |  | 34 (17.6) ^*^ | 41 (21.2) | 118 (61.1) |
| Junior high school | 51 (39.2) | 18 (13.9) | 61 (46.9) |  | 74 (43.8) | 35 (20.7) | 60 (35.5) |  | 139 (23.2) ^*^ | 127 (21.2) | 334 (55.7) ^*^ |
| Senior high school | 30 (32.6) | 25 (27.2) | 37 (40.2) |  | 54 (49.5) | 18 (16.5) | 37 (33.9) |  | 71 (22.6) ^*^ | 65 (20.7) | 178 (56.7) ^*^ |
| Junior college or above | 22 (28.6) | 15 (19.5) | 40 (52.0) |  | 16 (47.1) | 5 (14.7) | 13 (38.2) |  | 39 (31.0) | 33 (26.2) | 54 (42.9) |
| *P for trend* | *0.17* | *0.05* | *0.85* |  | *0.21* | *0.61* | *0.38* |  | ***0.02*** | *0.44* | ***0.01*** |
| BMI groups (kg/m^2^) |  |  |  |  |  |  |  |  |  |  |  |
| < 24.0 | 41 (33.6) | 21 (17.2) | 60 (49.2) |  | 62 (43.7) | 20 (14.1) | 60 (42.3) |  | 93 (24.5) ^*^ | 80 (21.1) | 206 (54.4) |
| 24.0~27.9 | 70 (36.7) | 36 (18.9) | 85 (44.5) |  | 77 (47.8) | 33 (20.5) | 51 (31.7) ^*^ |  | 144 (22.8) ^*^ | 139 (22.0) | 348 (55.2) ^*^ |
| ≥ 28.0 | 16 (30.8) | 9 (17.3) | 27 (51.9) |  | 24 (42.1) | 13 (22.8) | 20 (35.1) |  | 55 (21.5) ^*^ | 54 (21.1) | 147 (57.4) ^*^ |
| *P for trend* | *0.92* | *0.89* | *1.00* |  | *0.94* | *0.10* | *0.16* |  | *0.36* | *0.96* | *0.46* |
| WC groups (cm) |  |  |  |  |  |  |  |  |  |  |  |
| ≤ 90 | 82 (32.7) | 48 (19.1) | 121 (48.2) |  | 103 (43.5) ^*^ | 48 (20.3) | 86 (36.3) ^*^ |  | 153 (24.0) ^*^ | 132 (20.7) | 352 (55.3) ^*^ |
| > 90 | 43 (38.1) | 17 (15.0) | 53 (46.9) |  | 60 (48.8) | 18 (14.6) | 45 (36.6) |  | 139 (22.1) ^*^ | 141 (22.4) | 349 (55.5) ^*^ |
| *P for trend* | *0.32* | *0.35* | *0.82* |  | *0.34* | *0.19* | *0.96* |  | *0.42* | *0.46* | *0.94* |
| **Women** | 139 (33.3) | 84 (20.1) | 195 (46.7) |  | 166 (49.4) ^*^ | 68 (20.2) | 102 (30.4) ^*^ |  | 454 (31.5) ^*^ | 306 (21.2) | 683 (47.3) ^*^ |
| Age group (years) |  |  |  |  |  |  |  |  |  |  |  |
| 35~44 | 10 (35.7) | 2 (7.1) | 16 (57.1) |  | 8 (66.7) | 1 (8.3) | 3 (25.0) |  | 5 (22.7) | 4 (18.2) | 13 (59.1) |
| 45~54 | 21 (25.3) | 18 (21.7) | 44 (53.0) |  | 31 (42.5) | 15 (20.6) | 27 (37.0) |  | 26 (22.2) ^*^ | 19 (16.2) | 72 (61.5) ^*^ |
| 55~64 | 34 (37.8) | 20 (22.2) | 36 (40.0) |  | 60 (42.6) | 33 (23.4) | 48 (34.0) |  | 194 (31.3) ^*^ | 140 (22.6) | 285 (46.0) ^*^ |
| 65~74 | 74 (34.1) | 44 (20.3) | 99 (45.6) |  | 67 (60.9) ^*^ | 19 (17.3) | 24 (21.8) ^*^ |  | 229 (33.4) ^*^ | 143 (20.8) | 313 (45.7) ^*^ |
| *P for trend* | *0.45* | *0.43* | *0.18* |  | *0.07* | *0.92* | *0.06* |  | ***0.02*** | *0.65* | ***0.01*** |
| Educational level |  |  |  |  |  |  |  |  |  |  |  |
| Primary school or below | 79 (35.3) | 49 (21.9) | 96 (42.9) |  | 81 (58.7) ^*^ | 28 (20.3) | 29 (21.0) ^*^ |  | 100 (27.9) ^*^ | 77 (21.5) | 181 (50.6) ^*^ |
| Junior high school | 34 (30.6) | 20 (18.0) | 57 (51.4) |  | 57 (44.2) | 26 (20.2) | 46 (35.7) |  | 184 (31.1) ^*^ | 122 (20.7) | 286 (48.3) ^*^ |
| Senior high school | 18 (30.0) | 11 (18.3) | 31 (51.7) |  | 23 (39.0) | 10 (17.0) | 26 (44.1) |  | 109 (34.3) | 67 (21.1) | 142 (44.7) |
| Junior college or above | 6 (35.3) | 2 (11.8) | 9 (52.9) |  | 5 (50.0) | 4 (40.0) | 1 (10.0) |  | 24 (41.4) | 15 (25.9) | 19 (32.8) |
| *P for trend* | *0.49* | *0.25* | *0.11* |  | ***0.01*** | *0.75* | ***0.01*** |  | ***0.02*** | *0.75* | ***0.01*** |
| BMI groups (kg/m^2^) |  |  |  |  |  |  |  |  |  |  |  |
| < 24.0 | 53 (33.5) | 27 (17.1) | 78 (49.4) |  | 64 (48.5) ^*^ | 27 (20.5) | 41 (31.1) ^*^ |  | 174 (34.0) ^*^ | 100 (19.5) | 238 (46.5) ^*^ |
| 24.0~27.9 | 59 (36.0) | 34 (20.7) | 71 (43.3) |  | 78 (55.7) ^*^ | 24 (17.1) | 38 (27.1) ^*^ |  | 187 (31.3) ^*^ | 126 (21.1) | 284 (47.6) ^*^ |
| ≥ 28.0 | 27 (28.1) | 23 (24.0) | 46 (47.9) |  | 24 (37.5) | 17 (26.6) | 23 (35.9) |  | 93 (27.8) ^*^ | 80 (24.0) | 161 (48.2) |
| *P for trend* | *0.47* | *0.18* | *0.69* |  | *0.34* | *0.49* | *0.67* |  | *0.06* | *0.13* | *0.61* |
| WC groups (cm) |  |  |  |  |  |  |  |  |  |  |  |
| ≤ 80 | 58 (36.0) | 26 (16.2) | 77 (47.8) |  | 66 (52.8) ^*^ | 24 (19.2) | 35 (28.0) ^*^ |  | 136 (33.5) ^*^ | 85 (20.9) | 185 (45.6) ^*^ |
| > 80 | 80 (31.6) | 58 (22.9) | 115 (45.5) |  | 100 (47.4) ^*^ | 44 (20.9) | 67 (31.8) ^*^ |  | 318 (30.7) ^*^ | 221 (21.3) | 498 (48.0) ^*^ |
| *P for trend* | *0.35* | *0.09* | *0.64* |  | *0.34* | *0.72* | *0.47* |  | *0.30* | *0.88* | *0.40* |

Data was presented as count (percentage).

Glycemic control referring to PFG less than 7.0 mmol/L.

^*^ P < 0.0167 in comparison with previous survey;

Abbreviations: FPG, fasting plasma glucose; BMI, body mass index; WC, waist circumference.

**Supplementary Table 4** Glycemic control rates by HbA1c level among diagnosed diabetes patients

|  | The 2009 survey | | |  | The 2017 survey | | |
| --- | --- | --- | --- | --- | --- | --- | --- |
|  | <7.0 % | 7.0~8.0 % | >8.0 % |  | <7.0% | 7.1~8.0% | >8.0% |
| **All subjects** | 374 (54.5) | 138 (20.1) | 174 (25.4) |  | 1,126 (42.1) ^*^ | 751 (28.1) ^*^ | 798 (29.8) ^*^ |
| **Men** | 187 (52.4) | 61 (17.1) | 109 (30.5) |  | 500 (40.1) ^*^ | 344 (27.6) ^*^ | 404 (32.4) |
| Age group (years) |  |  |  |  |  |  |  |
| 35~44 | 8 (42.1) | 2 (10.5) | 9 (47.4) |  | 11 (33.3) | 8 (24.2) | 14 (42.4) |
| 45~54 | 55 (51.9) | 22 (20.8) | 29 (27.4) |  | 56 (38.9) | 45 (31.3) | 43 (29.9) |
| 55~64 | 65 (47.5) | 21 (15.3) | 51 (37.2) |  | 184 (37.4) ^*^ | 135 (27.4) ^*^ | 173 (35.2) |
| 65~74 | 59 (62.1) | 16 (16.8) | 20 (21.1) |  | 249 (43.0) ^*^ | 156 (26.9) ^*^ | 174 (30.1) |
| *P for trend* | *0.10* | *0.79* | *0.11* |  | *0.08* | *0.58* | *0.20* |
| Educational level |  |  |  |  |  |  |  |
| Primary school or below | 22 (44) | 11 (22) | 17 (34) |  | 72 (39) | 55 (29) | 60 (32) |
| Junior high school | 85 (51) | 30 (18) | 51 (31) |  | 231 (39) ^*^ | 163 (27) ^*^ | 200 (34) |
| Senior high school | 62 (58) | 16 (15) | 29 (27) |  | 116 (38) ^*^ | 86 (28) ^*^ | 107 (35) |
| Junior college or above | 18 (53) | 4 (12) | 12 (35) |  | 62 (50) | 34 (27) | 29 (23) |
| *P for trend* | *0.18* | *0.16* | *0.77* |  | *0.15* | *0.74* | *0.24* |
| BMI groups (kg/m^2^) |  |  |  |  |  |  |  |
| < 24.0 | 71 (50.4) | 20 (14.2) | 50 (35.5) |  | 149 (39.8) ^*^ | 105 (28.1) ^*^ | 120 (32.1) |
| 24.0~27.9 | 89 (56.0) | 30 (18.9) | 40 (25.2) |  | 253 (40.7) ^*^ | 167 (26.9) ^*^ | 201 (32.4) |
| ≥ 28.0 | 27 (49.1) | 11 (20.0) | 17 (30.9) |  | 98 (38.7) | 72 (28.5) | 83 (32.8) |
| *P for trend* | *0.84* | *0.25* | *0.24* |  | *0.83* | *0.97* | *0.85* |
| WC groups (cm) |  |  |  |  |  |  |  |
| ≤ 90 | 121 (51.7) | 41 (17.5) | 72 (30.8) |  | 257 (40.9) ^*^ | 168 (26.7) ^*^ | 204 (32.4) |
| > 90 | 66 (54.6) | 20 (16.5) | 35 (28.9) |  | 243 (39.3) ^*^ | 176 (28.4) ^*^ | 200 (32.3) |
| *P for trend* | *0.61* | *0.81* | *0.72* |  | *0.56* | *0.50* | *0.96* |
| **Women** | 187 (56.8) | 77 (23.4) | 65 (19.8) |  | 626 (43.9) ^*^ | 407 (28.5) | 394 (27.6) ^*^ |
| Age group (years) |  |  |  |  |  |  |  |
| 35~44 | 7 (58.3) | 3 (25.0) | 2 (16.7) |  | 7 (31.8) | 8 (36.4) | 7 (31.8) |
| 45~54 | 37 (52.1) | 20 (28.2) | 14 (19.7) |  | 40 (33.9) ^*^ | 33 (28.0) | 45 (38.1) ^*^ |
| 55~64 | 77 (55.4) | 31 (22.3) | 31 (22.3) |  | 277 (45.6) ^*^ | 170 (28.0) | 161 (26.5) |
| 65~74 | 66 (61.7) | 23 (21.5) | 18 (16.8) |  | 302 (44.5) ^*^ | 196 (28.9) | 181 (26.7) ^*^ |
| *P for trend* | *0.27* | *0.36* | *0.69* |  | *0.11* | *0.97* | *0.08* |
| Educational level |  |  |  |  |  |  |  |
| Primary school or below | 80 (61.1) | 34 (26.0) | 17 (13.0) |  | 148 (41.8) | 105 (29.7) | 101 (28.5) ^†^ |
| Junior high school | 64 (49.6) | 31 (24.0) | 34 (26.4) |  | 259 (44.2) | 166 (28.3) | 161 (27.5) |
| Senior high school | 35 (59.3) | 10 (16.9) | 14 (23.7) |  | 143 (45.1) ^*^ | 85 (26.9) | 89 (28.1) |
| Junior college or above | 8 (80.0) | 2 (20.0) | 0 (0.0) |  | 26 (46.4) | 17 (30.4) | 13 (23.2) |
| *P for trend* | *0.94* | *0.21* | *0.22* |  | *0.34* | *0.59* | *0.61* |
| BMI groups (kg/m^2^) |  |  |  |  |  |  |  |
| < 24.0 | 73 (57.5) | 32 (25.2) | 22 (17.3) |  | 232 (45.5) ^*^ | 140 (27.5) | 138 (27.1) ^*^ |
| 24.0~27.9 | 86 (61.4) | 32 (22.9) | 22 (15.7) |  | 265 (45.0) ^*^ | 160 (27.2) | 164 (27.8) ^*^ |
| ≥ 28.0 | 28 (45.2) | 13 (21.0) | 21 (33.9) |  | 129 (39.3) | 107 (32.8) | 92 (28.1) |
| *P for trend* | *0.22* | *0.50* | ***0.03*** |  | *0.10* | *0.14* | *0.74* |
| WC groups (cm) |  |  |  |  |  |  |  |
| ≤ 80 | 78 (64.5) | 26 (21.5) | 17 (14.1) |  | 198 (48.9) ^*^ | 107 (26.4) | 100 (24.7) ^*^ |
| > 80 | 109 (52.4) | 51 (24.5) | 48 (23.1) |  | 428 (41.9) ^*^ | 300 (29.4) | 294 (28.8) |
| *P for trend* | ***0.03*** | *0.53* | ***<0.05*** |  | ***0.02*** | *0.27* | *0.12* |

Data was presented as count (percentage).

Glycemic control referring to HbA1c lees than 7.0%.

^*^ P<0.05 in comparison with previous survey.

Abbreviations: HbA1c, hemoglobin A1c; BMI, body mass index; WC, waist circumference.


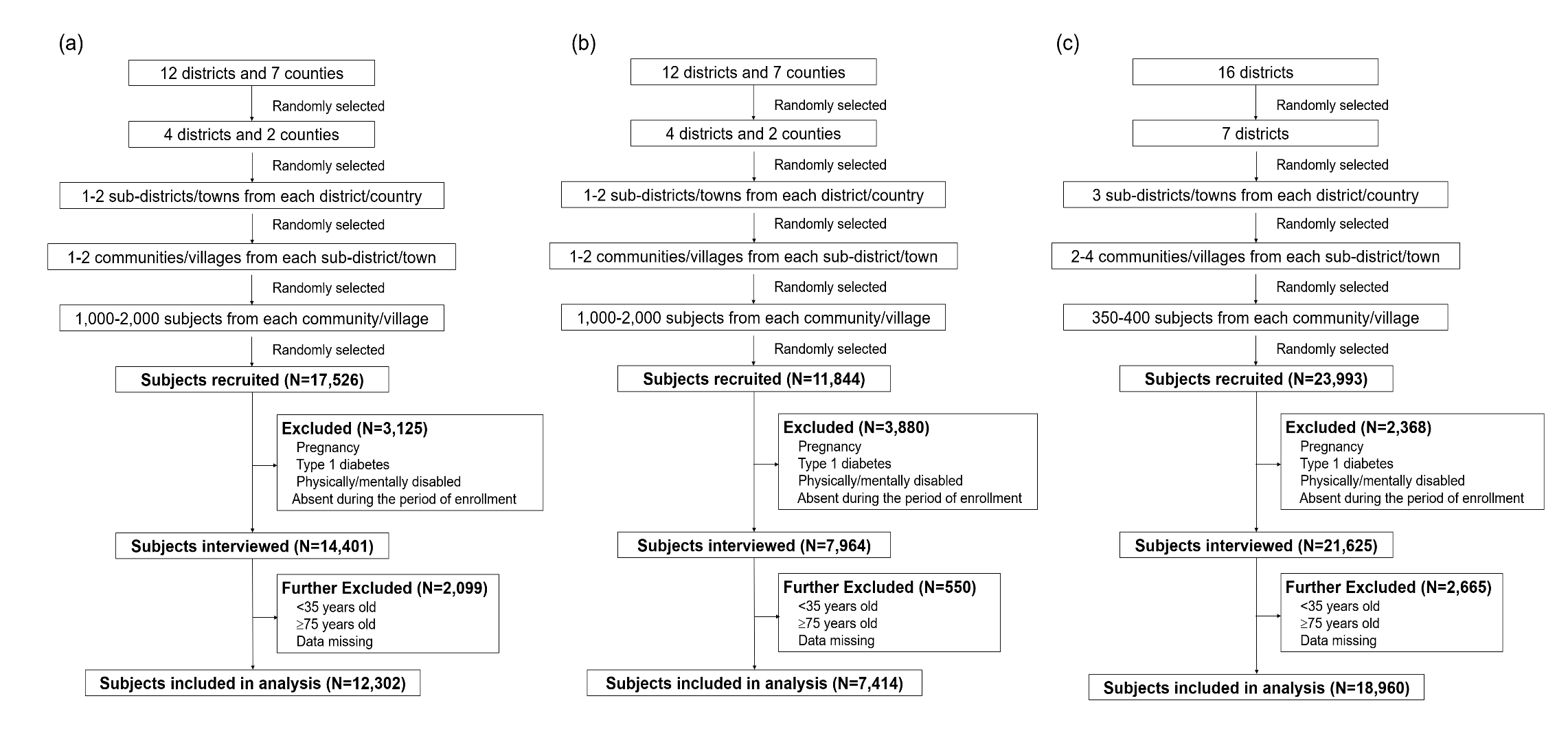


**Supplementary figure 1** Flow chart of participant recruitment in the 2002-03 (a), 2009 (b), and 2017 (c) survey.


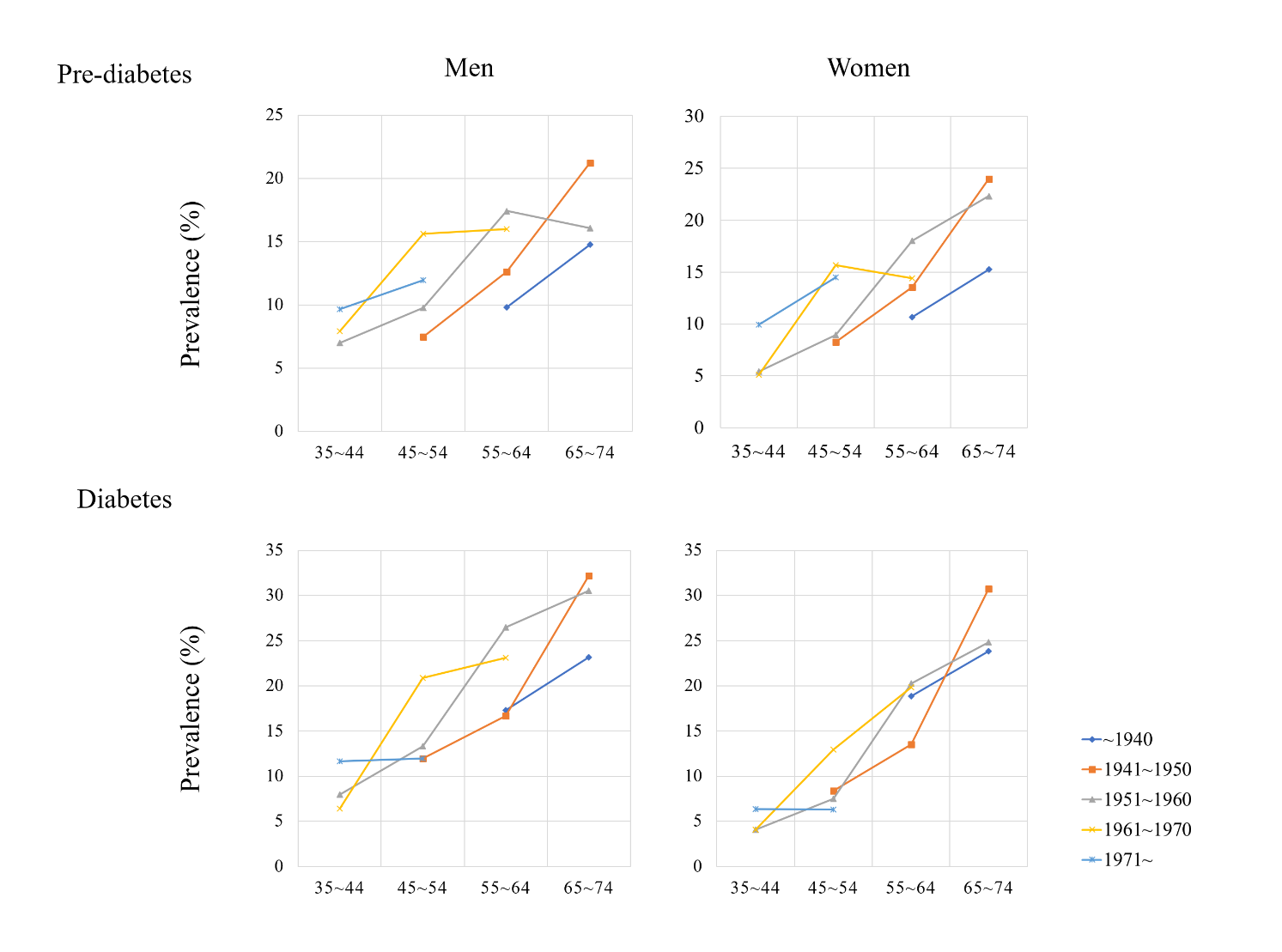


**Supplementary figure 2** Prevalence of diabetes and prediabetes by birth year and age group among Chinese men and women in 2002-03, 2009 and 2017.
